# *OsNAR2.1* induced endogenous nitrogen concentration variation affects transcriptional expression of miRNAs in rice

**DOI:** 10.3389/fpls.2023.1093676

**Published:** 2023-02-24

**Authors:** Yong Zhang, Xiaoru Fan, Yulong Wang, Pulin Kong, Ling Zhao, Xiaorong Fan, Yadong Zhang

**Affiliations:** ^1^ Institute of Food Crops, Jiangsu Academy of Agricultural Sciences, Jiangsu High Quality Rice Research and Development Center, Nanjing Branch of China National Center for Rice Improvement, Nanjing, China; ^2^ School of Chemistry and Life Science, Anshan Normal University, Anshan, China; ^3^ State Key Laboratory of Crop Genetics and Germplasm Enhancement, Key Laboratory of Plant Nutrition and Fertilization in Low-Middle Reaches of the Yangtze River, College of Resources and Environmental Sciences, Nanjing Agricultural University, Nanjing, China; ^4^ Zhongshan Biological Breeding Laboratory, Nanjing, China

**Keywords:** nitrogen concentration, OsNAR2.1, miRNA, rice, previously unidentified miRNA

## Abstract

The studies of rice nitrogen concentration on the expression of miRNA so far are mostly limited to the exogenous nitrogen, leaving the effect of endogenous nitrogen largely unexplored. OsNAR2.1 is a high-affinity nitrate transporter partner protein which plays a central role in nitrate absorption and translocation in rice. The expression of *OsNAR2.1* could influence the concentration of the endogenous nitrogen in rice. We showed that the expression and production of miRNA in rice can be influenced by manipulating the endogenous nitrogen concentration *via OsNAR2.1* transgenic lines. The small RNA content, particularly 24 nucleotides small RNA, expressed differently in two transgenic rice lines (nitrogen efficient line with overexpression of *OsNAR2.1* (Ov199), nitrogen-inefficient line with knockdown *OsNAR2.1* by RNAi (RNAi)) compared to the wild-type (NP). Comparative hierarchical clustering expression pattern analysis revealed that the expression profiles of mature miRNA in both transgenic lines were different from NP. Several previously unidentified miRNAs were identified to be differentially expressed under different nitrogen concentrations, namely miR1874, miR5150, chr3-36147, chr4-27017 and chr5-21745. In conclusion, our findings suggest that the level of endogenous nitrogen concentration variation by overexpression or knockdown *OsNAR2.1* could mediate the expression pattern and intensity of miRNA in rice, which is of high potential to be used in molecular breeding to improve the rice responses towards nitrogen utilization.

## Introduction

MicroRNAs (miRNA) are endogenous non-coding RNA molecules with about 21 nt in length, which typically suppress target gene expression at post-transcriptional and translational levels (Bartel, 2004; Brodersen et al., 2008; Yang et al., 2013). Recent studies suggest that miRNAs also take part in epigenetic control as regulators to modulate genome-wide epigenetic status. Mature miRNAs are derived from single-stranded hairpin precursors (pri-miRNA) through two cleavage steps by Dicer-LIKE 1 enzyme. The resulting miRNAs associate with argonaute proteins to form RNA-induced silencing complex (RISC) to target complementary mRNA, which will induce immediate mRNA degradation or suppress subsequent translational steps (Liu Q et al., 2014; Rogers and Chen, 2013). In plants, most miRNAs target binding sites located in open reading frames (ORFs) in an extensive sequence complementary manner, with a few exceptions to bind the non-coding untranslated regions (5’- and 3’-UTRs) of mRNAs (Allen et al., 2005; German et al., 2008).

Our understanding of the roles of miRNAs in plants has advanced tremendously over the past decade. In plants, miRNAs play crucial roles in almost all aspects of developmental and metabolic processes, including organ maturation (Juarez et al., 2004; Guo et al., 2005), hormone signaling (Liu et al., 2009) and plant development (Achard et al., 2004). In addition, miRNAs have been described to be involved in the biotic (microbial and viral pathogenesis) and abiotic stress responses in plants (drought, salinity, heavy metal, chilling and nitrogen stresses) (Sullivan and Ganem, 2005; Navarro et al., 2006; Zhao et al., 2007; Zhao et al., 2009; Zhou et al., 2008; Huang et al., 2009; Fischer et al., 2013).

Recently, many miRNAs have been reported for their specific role in plants. To date, a total of 738 known rice miRNAs have been deposited in the miRBase21 database (Kozomara et al., 2019). Some of the deposited miRNAs from the seven miRNA families, including the miR156, miR157 and miR399, are found to be involved in regulating the nitrogen (N) use efficiency (NUE) in multiple parts of plants (Cai et al., 2012). In maize, more than forty miRNA families, such as miR164, miR167, miR399, etc., are reported to be link to the regulation of NUE (Xu et al., 2011; Trevisan et al., 2012). Also, many miRNAs have been revealed to be participated in the NUE in Arabidopsis (Zhao et al., 2011; Liang et al., 2012) and soybean (Valdés-López et al., 2010). Vidal et al. (2010) showed that nitrate and sucrose applications in *Arabidopsis* roots are modulated by specific transcripts of miRNAs based on the microarray analysis (Vidal et al., 2010). Findings from the same study has demonstrated that miR393/*AFB3* module acts as unique nitrate-responsive regulatory network to regulates root system architecture in response to the availability of internal and external nitrogen sources in Arabidopsis.

Previous studies have shown that overexpression of *OsNAR2.1*, a high-affinity nitrate transporter, can increase the nitrogen concentration, NUE and yield in rice (Chen et al., 2017). Silencing of *OsNAR2.1* reduced the rice NUE and nitrogen concentration (Yan et al., 2011). Besides, other N responsive miRNAs were also identified based on the changes of plant NUE to various exogenous nitrogen supplies (Xu et al., 2011; Trevisan et al., 2012). In this study, we investigated the expression pattern of several N-responsive miRNAs in rice by changing the endogenous nitrogen content of plants *via* overexpression and silencing of *OsNAR2.1*. The expression levels of the targeted miRNAs were altered in transgenic plants. Interestingly, several previously unidentified N-responsive miRNAs have also been identified that responded to the changes of rice NUE following the alterations of rice endogenous nitrogen content.

## Materials and methods

### Construction of vectors and rice transformation

The *OsNAR2.1* overexpression transgenic line (Ov199) used in this study, which was named as pUbi-*OsNAR2.1*, have been described in detailed in previous studies (Chi et al., 2011; Chen et al., 2017). Briefly, we amplified the *OsNAR2.1* ORF sequence from cDNA isolated from the *Oryza sativa* L. ssp. *Japonica* and ligated it into the expression vector containing the Ubi promoter. The expression construct was later transferred into Agrobacterium tumefaciens strain EHA105 by electro-poration, followed by transformation into the rice as described by Upadhyaya et al. (Fan et al., 2020). A previously described procedure was adopted to generate the *OsNAR2.1* RNAi line (RNAi) (Yan et al., 2011).

### Plant materials and growth conditions

In field experiments, the seedlings of *OsNAR2.1* overexpression line (Ov199), *OsNAR2.1* RNAi line (RNAi) and wild type (NP) were grown in plots at the Nanjing Agricultural University in Nanjing, Jiangsu. Soil chemical properties and fertilizer application details are given in (Upadhyaya et al., 2000).

In hydroponic experiments, the seeds were surface-sterilized and germinated in the International Rice Re-search Institute (IRRI) solution. The detailed nutrient composition and pH of IRRI have been reported previously (Chen et al., 2016). The lines of Ov199, RNAi and NP were grown in a greenhouse with IRRI solution for one week under 14 h light (30^°^C)/10 h dark (22^°^C) photoperiod and 60% relative humidity conditions. For different nitrogen conditions treatment experiments, the seedlings of NP were grown under nitrogen deficient conditions for three days, followed by treating with 0.125 mM NH_4_NO_3_ (LN), 1.25 mM NH_4_NO_3_ (NN) and 2.5 mM NH_4_NO_3_ (HN) for one week. The RNA samples of NP were extracted after the treatment.

### miRNA sequence

The pooled RNA samples extracted from the leaves, flowers and stems of rice obtained during the blooming stage were sent for sequencing services by the Kangchen Bio-tech Inc., China. The quality and quantity of RNA samples were analyzed by NanoDrop ND-1000 model. The quantified RNA was used to synthesize cDNA, followed by the ligation of 5’- and 3’-adapters to create the sequencing library. The cDNA samples were diluted to a final concentration of 8 pM prior to generate clusters on Illumina cBlot using TruSeq SR Cluster kit (#GD-402-4001, Illu-mina). Sequencing was performed on Illimina HiSeq 2000 using TruSeq Rapid SBS Kit (#FC-402-4001, Illumina), followed by data acquisition and processing sequence. The sequence data have been deposited in the NCBI GEO under accession number GSE224933.

### Novel miRNA prediction

All unmatched sequences from the full small RNA sequencing data to the known miRNA annotations were analyzed with miRDeep2 package (http://www.mdcberlin.de/en/research/research_teams/systems_biology_of_gene_regulatory_elements/projects/miRDeep/) to construct potential models of miRNA precursors by exploring the Dicer cleavage sites in order to predict the novel miRNAs (Zhang et al., 2020).

### miRNA quantitative real-time (qRT) PCR

The previously described protocols for total RNA isolation and concentration measurement were used with modifications (Friedländer et al., 2008; Yan et al., 2011). The synthesis of miRNA first-strand cDNA was performed with miRNA First-Strand cDNA Synthesis SuperMix (Vazyme, Co. R323-01, Nanjing, China), prior to the amplification of qRT-PCR products with the AceQ qPCR SYBR Green Master Mix kit (Vazyme Biotech Co. Q311-02, Nanjing China.) using a Step One Plus Real-Time PCR System (Applied Biosystems, Foster City, CA, USA). The details of primers for the PCR are shown in **Supplementary Table 5**.

### Statistical analysis of the data

The collected data were tabulated and analyzed for significant differences using the IBM SPSS Statistics 20 program and One-Way ANOVA, followed by Tukey’s test (*P* < 0.05).

## Results

### The roles of *OsNAR2.1* in the rice growth

**Figure 1:**
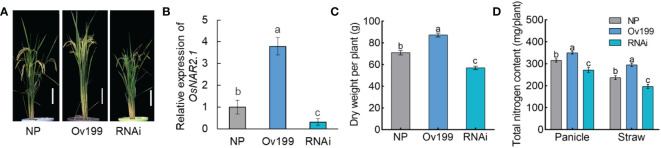
Characteristics of phenotype for miRNA sequencing plants. **(A)** Gross morphology of wild-type of NP, Ov199 and RNAi. Bar, 20 cm. **(B)** Real-time quantitative RT-PCR analysis of *OsNAR2.1* expression in NP, Ov199 and RNAi lines. Dry weight **(C)** and total nitrogen content **(D)** of NP, Ov199 and RNAi. Error bars: SD (n = 3). NP, the wild-type of *Oryza.Sativa* L.spp.*Japonica*. Ov199, *OsNAR2.1* overexpression transgenic line. RNAi, *OsNAR2.1* RNAi line. Significant differences between different lines are indicated by different letters (*P* < 0.05, one-way ANOVA).

In rice, OsNAR2.1 is a high affinity nitrate transporter partner protein that plays a critical role in the absorption of nitrate (Yan et al., 2011; Liu X et al., 2014). Previously, the transgenic rice plants p35S:*OsNAR2.1* and p*OsNAR2.1*:*OsNAR2.1* were found to promote plant growth and increase the N concentration, N content and yield of rice (Chen et al., 2017; Chen et al., 2020). Also, overexpression of *OsNAR2.1* was found to enhance drought tolerance and grain yield under drought stress conditions in rice (Chen et al., 2019). In order to explore the regulatory mechanism of *OsNAR2.1* in rice growth, the agronomic traits of the pUbi : *OsNAR2.1* overexpression line (Ov199), *OsNAR2.1* RNAi suppression line (RNAi) and the non-transgenic line (NP) were investigated (**Figure 1**). The plant height was substantial increased in Ov199 lines, but was reduced in RNAi lines as compared to NP (**Figures 1A, B**). In addition, the study reported that the seeds weight per panicle, the number of seeds per panicle, the seed setting rate and the yield per plant in Ov199 were significantly increased by 57.3%, 63.8%, 20.2% and 35.0%, respectively than the NP line. Similar agronomic traits were significantly reduced by 24.8%, 27.0%, 32.1% and 51.1%, respectively in RNAi line as compared to the NP (Liu X et al., 2014). And the dry weight of Ov199 was increased compared with NP, and reduced in RNAi lines (**Figure 1C**). Total N content of Ov199 was substantial increased in Ov199 lines compared with NP, but opposite in RNAi lines (**Figure 1D**). Hence, our findings strongly suggest that *OsNAR2.1* plays a vital role in the rice growth and the output in rice production.

### The effects of *OsNAR2.1* level on the expression of miRNAs in rice

**Figure 2:**
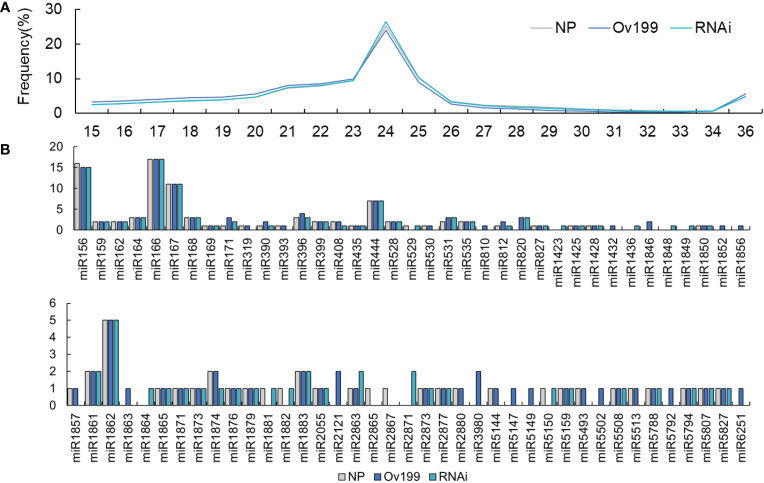
Number of identified members of each conserved miRNA families in NP, Ov199 and RNAi. **(A)** Size distribution of the small RNA in NP, Ov199 and RNAi lines. **(B)** Number of identified members of each conserved miRNA families in NP, Ov199 and RNAi.

In order to explore whether the overexpression or silencing of *OsNAR2.1* affect the expression of small RNAs, we performed small RNA sequencing by using Ov199, RNAi and NP lines. After excluding the low-quality reads and adaptors, the align clean reads of the small RNAs for the Ov199 and RNAi lines were found to be higher in quantity than those of NP (**Supplementary Table 1**). Further analysis of the retained small RNA from all lines have found that the prominent sizes of the small RNAs left were 21-25 in length (**Figure 2A**). The 24 nt small RNAs accounted for 25.21%, 24.05%, 26.42% of total small RNAs in NP, Ov199 and RNAi line, respectively. The proportion of 24 nt small RNA in Ov199 was lower than that of NP and RNAi (**Figure 2A**). Meanwhile the 21 nt small RNAs accounted for 7.31%, 8.02%, and 7.41% of total small RNAs in NP, Ov199 and RNAi, respectively. The proportion of 21 nt small RNAs in Ov199 was higher than that of NP and RNAi (**Figure 2A**).

The microarrays analysis of miRNAs from the three experimental lines revealed that one hundred sixty-five known miRNAs belong to the seventy-six miRNA families were differentially expressed (**Figure 2B**). The number of known miRNA family members varies greatly on the basis of the differences in endogenous nucleotides. The miR166 family has the highest number of members, which can be classified into seventeen different miRNA families (**Figure 2B**). Besides, the miR156, miR167, miRNA444 and miR1862 families are groups that contain 5-15 members. The rest of the known miRNAs fall into twenty-three miRNA families each containing 2-4 members and forty single-membered miRNA families (**Figure 2B**).

**Figure 3:**
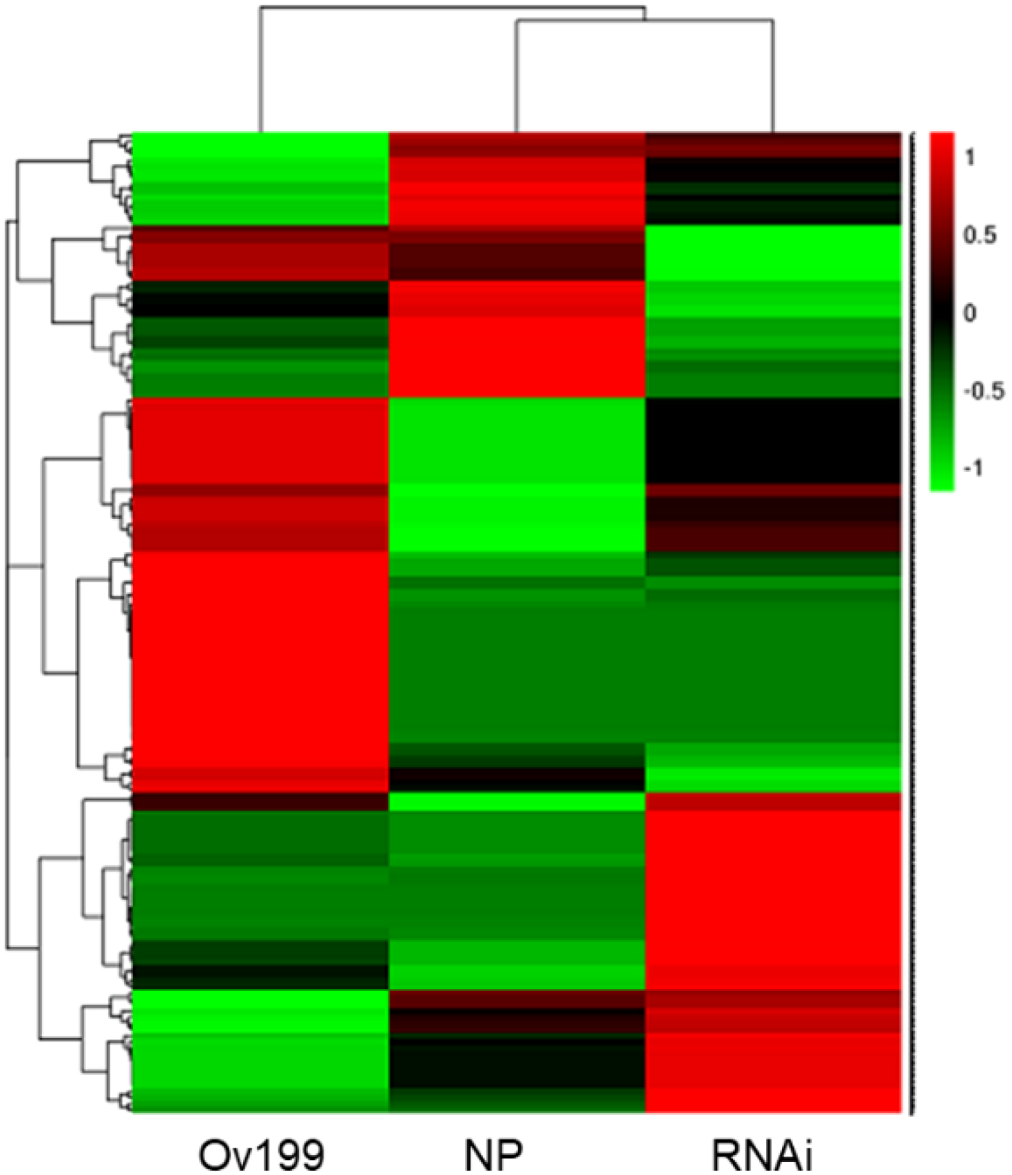
Hierarchical clustering and expression patterns of known miRNA in NP, Ov199 and RNAi.

The expression abundance of miRNAs in NP, Ov199 and RNAi was different based on the normalized transcript per million (TPM) analysis of miRNA data. Through hierarchical clustering and expression analysis, we found that the expression level of 45% of miRNAs in Ov199, 52% of miRNAs in RNAi and 35% of miRNAs in NP from was upregulated (**Figure 3**; **Supplementary Table 2**). Among the known miRNAs, miR1876 was the only miRNA upregulated/expressed in all experimental lines. The number of transcription units for NP, Ov199 and RNAi were 36,075, 32,893 and 43,939, respectively. The expression level was highest in the miR166 family, followed by miR167, miR444, miR168 and the remaining miRNA families (**Supplementary Table 2**). Some miRNAs grouped under the same family were found to be differentially expressed in different experimental lines (**Supplementary Table 2**). For example, miR156a has the lowest expression in NP, but the miRNA with the lowest expression in Ov199 and RNAi was miR156b-3p and miR156j-3p, respectively (**Supplementary Table 2**).

### *OsNAR2.1* regulates multiple miRNA targets

**Figure 4:**
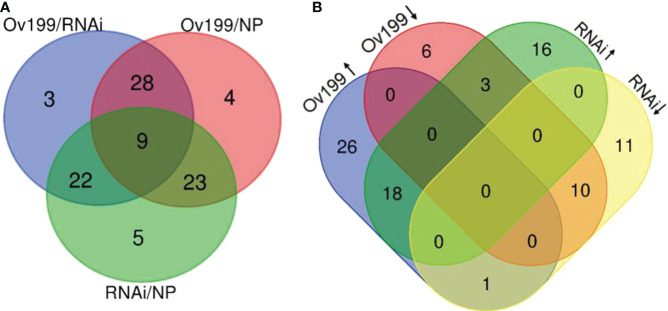
Analysis of significant difference miRNA between NP, Ov199 and RNAi. **(A)** Venn diagram of unique and shared miRNA with significant difference expression between Ov199 and NP, RNAi and NP, and Ov199 and RNAi. **(B)** Venn diagram of unique and shared miRNA with significant difference expression between upregulated and downregulated miRNA in Ov199 and RNAi compared with NP.

In order to identify the N-responsive miRNAs, we comparatively analyzed the expression of miRNAs from all the experimental lines. The comparative results from three comparison groups (i.e., Ov199/RNAi, Ov199/NP, RNAi/NP) showed that there were 62, 64 and 59 differentially expressed miRNAs in each respective group with *P <*0.05 significant difference (**Figure 4A**; **Table S2**). Nine out of all differentially expressed miRNA were found to be significantly expressed in all comparison groups, while majority of them only altered in two comparison groups. There were 3, 4 and 5 miRNAs with significant different levels exclusively expressed in group Ov199/RNAi, Ov199/NP and RNAi/NP, respectively (**Figure 4A**).

**Figure 5:**
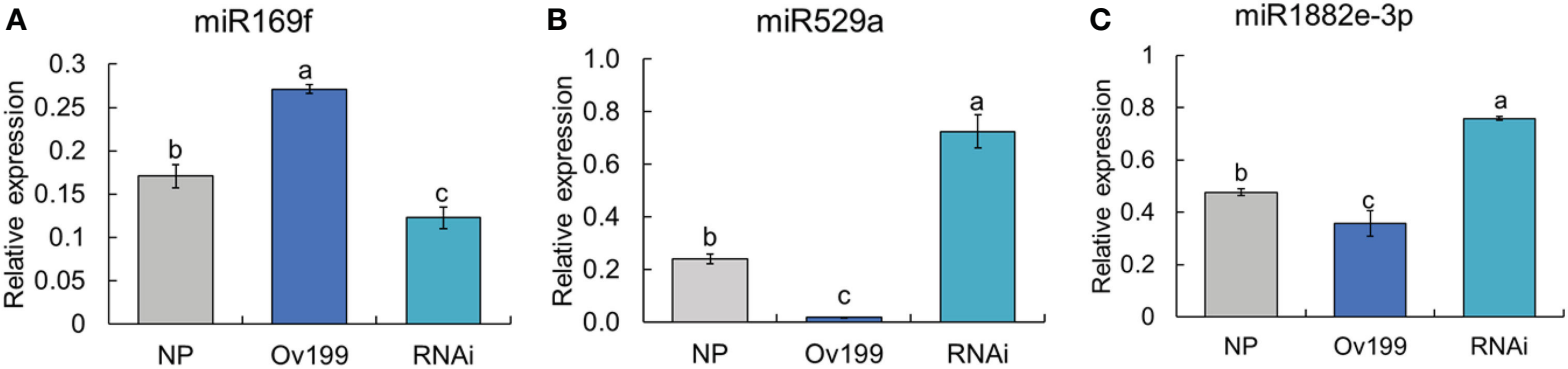
The results of RT-qPCR of miRNAs in NP, Ov199 and RNAi. **(A)** Expression of miR169f in NP, Ov199 and RNAi. **(B)** Expression of miR529a in NP, Ov199 and RNAi. **(C)** Expression of miR1882e-3p in NP, Ov199 and RNAi. Error bars: SD (n = 4). Significant differences between different lines are indicated by different letters (*P* < 0.05, one-way ANOVA).

Differential expression analysis of miRNA between transgenic lines and the control (NP) showed that only the peak expression of miR169f in Ov199 and RNAi was consistent with the changes in *OsNAR2.1* expression and total nitrogen concentration (**Figure 4B**; **Supplementary Table 2**). The expression peak of miR529a, miR1882e-3p and miR5150-5p in Ov199 and RNAi were opposite to the expression level of *OsNAR2.1* and total N concentration (**Figure 4B**; **Supplementary Table 2**). The expression patterns of miR169f, miR529a and miR1882e-3p were consistent with the sequencing results as verified by RT-PCR (**Figure 5**). The expression level of miR169f was significantly upregulated in Ov199 and downregulated in RNAi as compared to the NP (**Figure 5A**). In contrary, the expression of miR529a and miR188e-3p were significantly downregulated in Ov199 and upregulated in RNAi (**Figures 5B, C**). Therefore, *OsNAR2.1* changes the nitrogen concentration and content in rice and maybe affect the expression of multiple miRNAs.

### Analysis of novel miRNAs in transgenic *OsNAR2.1* lines

**Figure 6:**
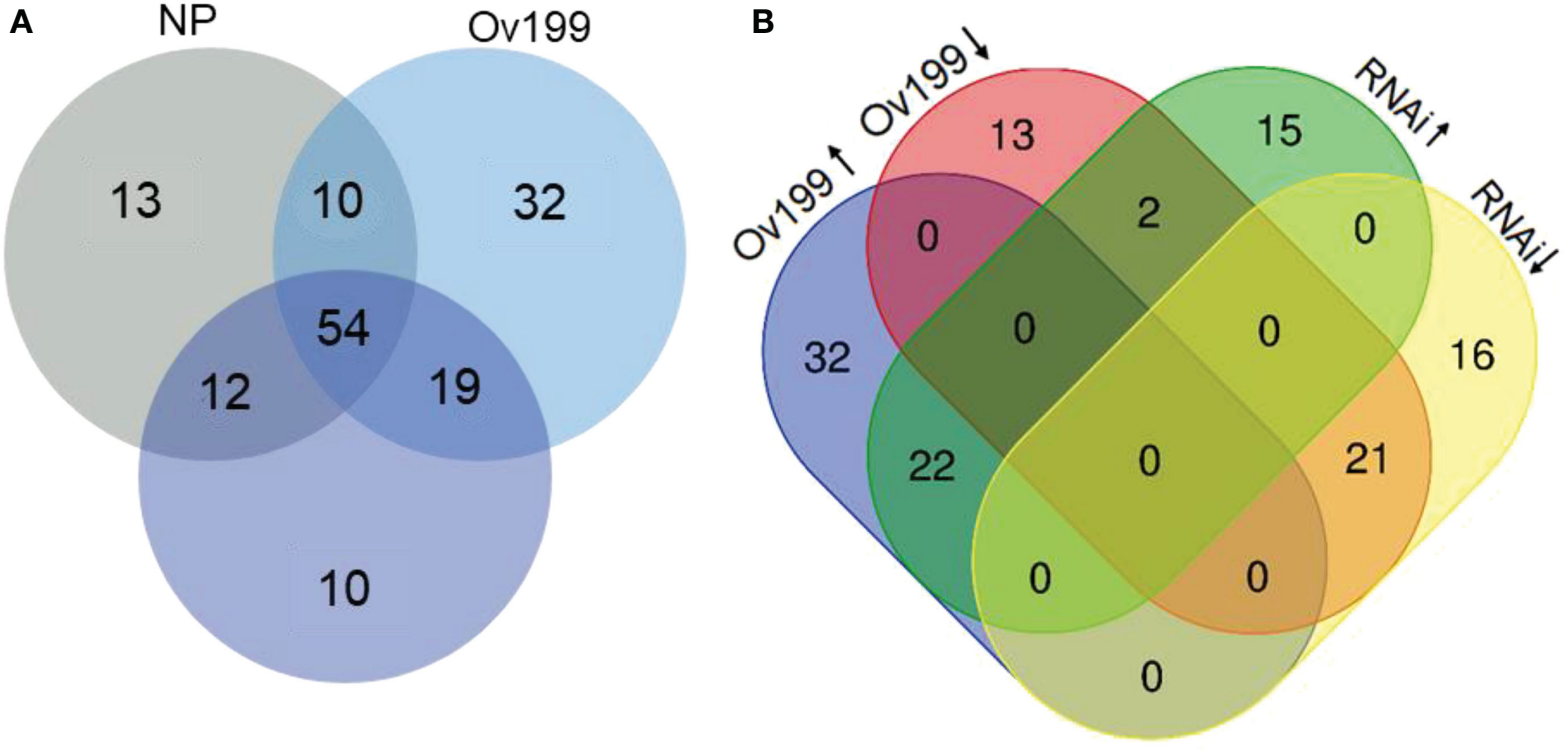
Analysis of significant difference novel miRNA between NP, Ov199 and RNAi. **(A)** Venn diagram of unique and shared miRNA in NP, Ov199 and RNAi. **(B)** Venn diagram of unique and shared miRNA with significant difference expression between upregulated and downregulated miRNA in Ov199 and RNAi compared with NP.

Identification of novel miRNAs from the transgenic *OsNAR2.1* lines was performed with miRDeep analysis package. A total of 150 novel miRNAs were identified from all experimental lines with an approximate length of 17-23 nt, of which the most common length falls in 24 nt (**Supplementary Table 3**). There were 13, 32 and 10 specific novel miRNAs that only expressed in NP, Ov199 and RNAi, respectively; while 54 novel miRNAs were found in all the three lines (**Figure 6A**). Besides, there were 64 novel miRNAs found to be overlapped in group NP/Ov199, whereas 66 and 73 novel miRNAs overlapped in group NP/RNAi and Ov199/RNAi, respectively (**Figure 6A**). Compared with NP, 22 novel miRNAs were upregulated in Ov199 but 21 of them were downregulated in RNAi (**Figure 6B**). By referring to the known miRNA families, the abundance of novel miRNAs was very low, and more than 75% of the novel miRNAs have less than 100 transcript units (**Supplementary Table 3**). There were only five novel miRNAs with high abundance of more than 1000 transcript units that is miR-novel-chr1-37617, miR-novel-chr1-38364, miR-novel-chr1-43165, miR-novel-chr4-27017 and miR-novel-chr8-11525 (**Supplementary Table 3**). And the prediction target genes of miR-novel-chr1-38364, miR-novel-chr1-43165 and miR-novel-chr4-27017 are *LOC_Os09g3939.2*, *LOC_Os09g10820.1*, *LOC_Os08g9080.2*, respectively, and others no corresponding target gene (**Supplementary Table 4**). All these five highly abundance novel miRNAs expressed differentially in all the three lines. In general, the expression level of novel miRNAs was relatively downregulated in RNAi compared to the NP and Ov199 (**Supplementary Table 3**). The Ov199 line has the highest number of novel miRNAs (76.7%) from its total number of miRNAs, which was much higher than the NP (59.3%) and RNAi (63.3%) lines. The results implied that the overexpression of *OsNAR2.1* gene in Ov199 line might help to promote the expression of novel miRNA in the rice plants.

**Figure 7:**
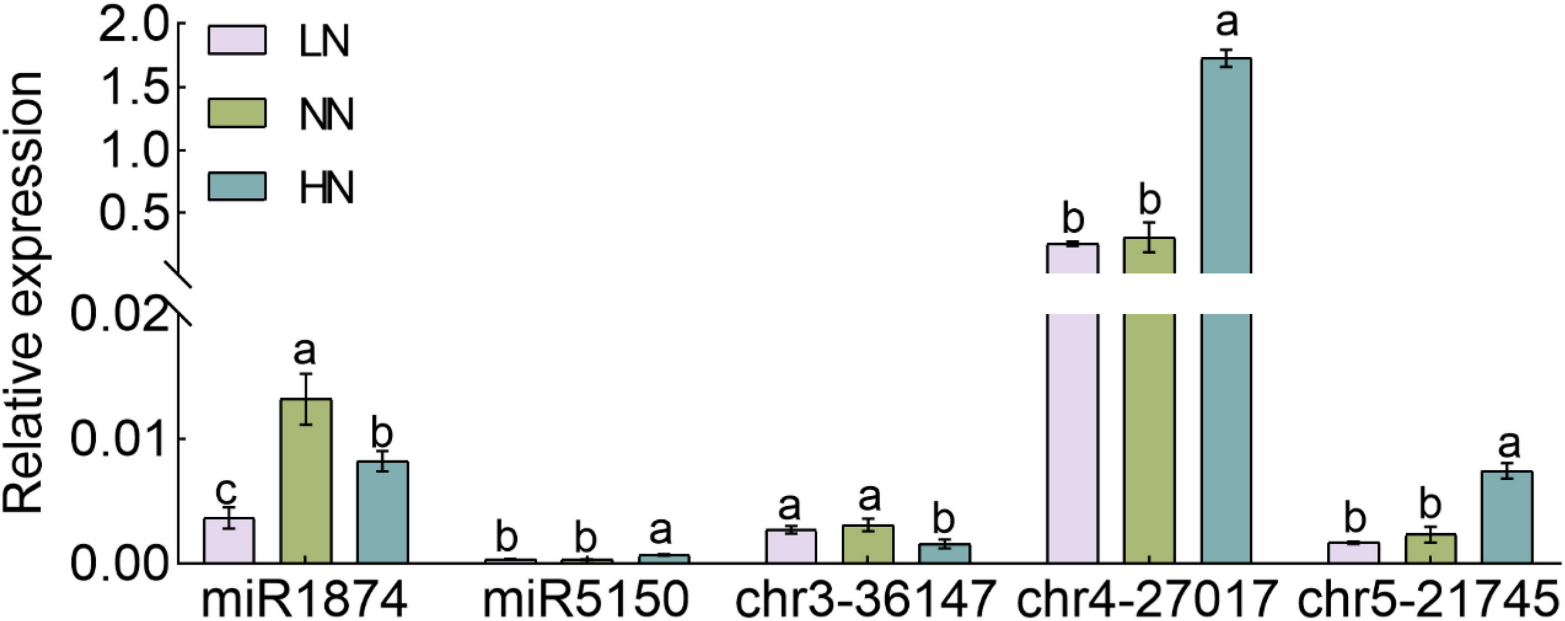
The expression of novel miRNA under different nitrogen treatment. Seedlings of rice cultivated, Nipponbare, in hydroponics for three weeks and were grown under nitrogen deficient conditions for three days, followed by treating with 0.125 mM NH_4_NO_3_ (LN), 1.25 mM NH_4_NO_3_ (NN) and 2.5 mM NH_4_NO_3_ (HN) for one week. The RNA samples of Nipponbare were extracted one week after the treatment. The expressions of miR1874, miR5150, chr3-36147, chr4-27017, chr5-21745 were taken. Error bars: SD (n = 3). Significant differences between different lines are indicated by different letters (*P* < 0.05, one-way ANOVA).

In order to examine the effects of different nitrogen concentrations in the regulation of miRNA expression, we performed miRNA expression verification on rice under high nitrogen (HN), low nitrogen (LN) and normal nitrogen (NN) treatments. A total of five miRNAs were selected as the candidates for the verification purposes that is miR1874, miR5150, chr3-36174, chr4-27017 and chr5-21745 (**Figure 7**). The results of qRT-PCR showed that the expression of miR1874 was inhibited in both HN and LN treatments as compared to the NN. The inhibition on the chr3-36174 was also observed in HN, but was not in LN (**Figure 7**). The expression of miR5150, chr4-27017 and chr5-21745 was up-regulated under HN treatment in relative to the NN, but was not significantly different under LN condition (**Figure 7**).

## Discussion

### Association of the roles of miRNAs and the N regulation in rice

The nitrogen concentration of rice can be altered with the expression of *OsNAR2.1*, which can result in the epigenetic changes in rice, including the expression pattern and intensity of miRNAs. It has been reported that miR169 in maize, common bean (Pharsalus vulgaris) and Arabidopsis responded to N starvation (Valdés-López et al., 2010; Xu et al., 2011; Zhao et al., 2011; Liang et al., 2012; Trevisan et al., 2012). Besides, the miR393/*AFB3* has been linked to the regulation of root system architecture in response to the internal and external N availability in *Arabidopsis* (Vidal et al., 2010). The lateral root growth is usually associated with the nitrate uptake, but the overexpression of miR393 was found to alter the primary and lateral root growth after nitrate application (Vidal et al., 2010). Recently, an auxin-related regulatory miR167/ARF8 module has been reported to regulate the ratio between emerging and initiating lateral roots in Arabidopsis. The cell-specific regulation of a transcriptional circuit mediates lateral root growth in response to the N *via* miRNA167 (Gifford et al., 2008). Furthermore, the expression of recently described miR529 was found to render some agronomic traits of rice, such as rice tiller number, panicle type and hydrogen peroxide resistance. The miR529 targets five SPL genes (*OsSPL2*, *OsSPL14*, *OsSPL16*, *OsSPL17* and *OsSPL18*), where the *OsSPL14* expression showed to be inversely correlated to the miR529a (Yue et al., 2017). The *OsSPL14* has been shown to improve the nitrogen uptake in rice with an expression pattern oppositely responses to the level of nitrogen supply (Srikanth et al., 2016; Wu et al., 2020). Studies shown that the endogenous N content and concentration of rice *via* transgenic *OsNAR2.1* lines to determine miRNAs that could be responding to the changes of endogenous N (**Figures 1**, **2**, **4**). Therefore, it is speculated that the expression peaks of these miRNAs might be correlated to the expression of *OsNAR2.1* and the nitrogen concentration in rice.

### Overexpression and silencing of *OsNAR2.1* gene affect the expression of a large number of N-responsive miRNAs

High-throughput sequencing approach has been using in many studies to analysis genome-wide miRNA expression in plants (Zhao et al., 2007; Chi et al., 2011; Liang et al., 2012). Apart from that, transgenic technology provides a permissible alternative for *in vivo* study to examine the effect of endogenous nitrogen concentration of rice towards the miRNA expression, which allows further attempts to improve the understanding between miRNA and the rice NUE. Nitrogen-deficient conditions cause plants to deplete N internally. As an emergency response, they regulate the ability to absorb and transport N by regulating the genes and small RNA expression. The report shown that during nitrification and absorption, miR393 is activated by the N signal transmitted, and effected the function of the phytohormone sensing organ auxin signaling F-box proteins (AFB3) (Bao et al., 2014; Sajjad et al., 2021). In maize plants deficient in N, six miRNAs were downregulated, including miR408, miR169, miR166, miR528*, and miR169* (Yang et al., 2019).

In our study, the number of miRNAs being expressed was highest in the nitrogen-efficient line (Ov199), followed by the nitrogen-inefficient line (RNAi) and the wild-type (NP), regardless of the known or novel miRNAs (**Supplementary Table 1**). Among the 159 known miRNAs, both transgenic lines have about the same number of the highest expressing miRNAs, i.e., 45% and 52% of known miRNAs in Ov199 and RNAi, respectively. Meanwhile, there was only 35% of the known miRNAs with the highest expression in NP. The results indicate that *OsNAR2.1* may play a role in the transgenic lines to alter the rice endogenous nitrogen environment, resulting in the differential expression pattern of a large number of miRNAs in rice.

### Discovery of novel miRNAs responsible for the NUE in Ov199 and RNAi lines

Previously, many known miRNAs have been reported to be involved in nitrogen related metabolisms in plants. The nitrogen concentration which was alter in plants may affect the expression of miRNA. More than 40 miRNAs, such as miR164, miR167, and miR399, are responsive towards nitrogen starvation in different parts of maize (Xu et al., 2011; Trevisan et al., 2012). The miRNAs from 7 families, including miR156, miR157, miR399, participate in the regulation of NUE in different parts of rice (Cai et al., 2012). A total of 19 miRNAs families, such as miR156, miR157, miR160, are involved in the regulation of NUE in Arabidopsis (Zhao et al., 2011; Liang et al., 2012). Also, there are 25 miRNAs in soybeans involved in the regulation of soybean NUE, such as miR396, miR164, miR168 (Valdés-López et al., 2010).

By comparing the miRNA sequencing data from all the experimental lines, we have identified four miRNAs (miR169f, miR529a, miR1882e-3p and miR5150-5p) expressed in a way that significantly inversed between the transgenic lines and the wild-type (**Figure 5**). Among them, miR169 has been reported to respond to the nitrogen deficient conditions in corn (Xu et al., 2011; Trevisan et al., 2012), soybean (Liang et al., 2012), and *Arabidopsis* (Zhao et al., 2011; Liang et al., 2012). The protein-coding gene *NFYA*, which is the target gene of miR169, encodes for the nitrate transporter proteins *NRT1.1* and *NRT2.1* as reported before (Zhao et al., 2011). MicroRNA529 (miR529) targets the *OsSPL14* gene by reducing its expression level with the increase of nitrogen supply, showing its important roles in nitrogen metabolism of rice (Srikanth et al., 2016; Wu et al., 2020). The results showed the involvement of miR169 and miR529 in rice NUE regulation. To date, there is no direct evidence to support the expression of miR1882e-3p and miR5150-5p being related to the NUE in rice. In addition, some miRNAs can respond to changes in endogenous nitrogen as well as changes in exogenous nitrogen, such as miR1874, miR5150, chr3-36147, chr4-27017 and chr5-21745 (**Figures 6**, **7**). By changing the NUE in rice, we can find novel mature miRNAs related to nitrogen, which will be useful for future research on NUE-related miRNAs.

In summary, the manipulation of rice endogenous nitrogen concentration through genetic modification can be an effective approach for the discovery of novel N-responsive miRNAs. Thus, the current research provides new insights for the detection of nitrogen related novel miRNAs.

## Data availability statement

Sequencing data supporting the findings of the article have been deposited in the NCBI Gene Expression Omnibus (GEO) under accession number GSE94319.

## Author contributions

Conceptualization, XroF. Data curation, YoZ, XruF, YW, PK and YaZ. Writing—original draft preparation, YoZ and XroF. Writing—review and editing, YoZ, XruF, YW, LZ and XroF. Visualization, YoZ. Supervision, XroF. Project administration, XroF. Funding acquisition, XroF and YoZ. All authors contributed to the article and approved the submitted version.

## References

[B1] Achard P. Herr A. Baulcombe D. C. Harberd N. P. (2004). Modulation of floral development by a gibberellin-regulated microRNA. Development 131, 3357–3365. doi: 10.1242/dev.01206 15226253

[B2] Allen E. Xie Z. Gustafson A. M. Carrington J. C. (2005). MicroRNA-directed phasing during trans-acting siRNA biogenesis in plants. Cell 121, 207–221. doi: 10.1016/j.cell.2005.04.004 15851028

[B3] Bao M. Bian H. Zha Y. Li F. Sun Y. Bai B.. (2014). MiR396a-mediated basic helix-loop-helix transcription factor bHLH74 repression acts as a regulator for root growth in arabidopsis seedlings. Plant Cell Physiol. 55, 1343–1353. doi: 10.1093/pcp/pcu058 24793750

[B4] Bartel D. P. (2004). MicroRNAs: Genomics, biogenesis, mechanism, and function. Cell 116, 281–297. doi: 10.1016/S0092-8674(04)00045-5 14744438

[B5] Brodersen P. Sakvarelidze-Achard L. Bruun-Rasmussen M. Dunoyer P. Yamamoto Y. Y. Sieburth L.. (2008). Widespread translational inhibition by plant miRNAs and siRNAs. Science 320, 1185–1190. doi: 10.1126/science.1159151 18483398

[B6] Cai H. M. Lu Y. G. Xie W. B. Zhu T. Lian X. M. (2012). Transcriptome response to nitrogen starvation in rice. J. Biosci. 37, 731–747. doi: 10.1007/s12038-012-9242-2 22922198

[B7] Chen J. G. Fan X. R. Qian K. Y. Zhang Y. Song M. Q. Liu Y.. (2017). p*OsNAR2.1*:*OsNAR2.1* expression enhances nitrogen uptake efficiency and grain yield in transgenic rice plants. Plant Biotechnol. J. 15, 1273–1283. doi: 10.1111/pbi.12714 28226420 PMC5595721

[B8] Chen J. G. Liu X. Q. Liu S. H. Fan X. R. Zhao L. M. Song M. Q.. (2020). Co-Overexpression of *OsNAR2.1* and *OsNRT2.3a* increased agronomic nitrogen use efficiency in transgenic rice plants. Front. Plant Sci. 11. doi: 10.3389/fpls.2020.01245 PMC7434940 32903417

[B9] Chen J. G. Qi T. T. Hu Z. Fan X. R. Zhu L. L. Iqbal M. F.. (2019). *OsNAR2.1* positively regulates drought tolerance and grain yield under drought stress conditions in rice. Front. Plant Sci. 10. doi: 10.3389/fpls.2019.00197 PMC6393350 30846998

[B10] Chen J. G. Zhang Y. Tan Y. W. Zhang M. Zhu L. Xu G. H.. (2016). Agronomic nitrogen-use efficiency of rice can be increased by driving *OsNRT2.1* expression with the *OsNAR2.1* promoter. Plant Biotechnol. J. 14, 1705–1715. doi: 10.1111/pbi.12531 26826052 PMC5066696

[B11] Chi X. Yang Q. Chen X. Wang J. Pan L. Chen M.. (2011). Identification and characterization of microRNAs from peanut (*Arachis hypogaea* l.) by high-throughput sequencing. PloS One 6 (11), e27530. doi: 10.1371/journal.pone.0027530 22110666 PMC3217988

[B12] Fan X. R. Chen J. G. Wu Y. F. Teo C. H. Xu G. H. Fan X. R. (2020). Genetic and global epigenetic modification, which determines the phenotype of transgenic rice? Int. J. Mol. Sci. 21, 1819. doi: 10.3390/ijms21051819 32155767 PMC7084647

[B13] Fischer J. J. Beatty P. H. Good A. G. Muench D. G. (2013). Manipulation of microRNA expression to improve nitrogen use efficiency. Plant Sci. 210, 70–81. doi: 10.1016/j.plantsci.2013.05.009 23849115

[B14] Friedländer M. R. Chen W. Adamidi C. Maaskola J. Einspanier R. Knespel S.. (2008). Discovering microRNAs from deep sequencing data using miRDeep. Nat. Biotechnol. 26, 407–415. doi: 10.1038/nbt1394 18392026

[B15] German M. A. Jeong D. H. Hetawal A. Luo S. Janardhanan P. Kannan V.. (2008). Global identification of microRNA-target RNA pairs by parallel analysis of RNA ends. Nat. Biotechnol. 26 (8), 941–946. doi: 10.1038/nbt1417 18542052

[B16] Gifford M. L. Dean A. Gutierrez A. R. Coruuzi M. G. Birnbaum D. K. (2008). Cell-specific nitrogen responses mediate developmental plasticity. Proc. Natl. Acad. Sci. U.S.A. 105, 803–808. doi: 10.1073/pnas.0709559105 18180456 PMC2206617

[B17] Guo H. S. Xie Q. Fei J. F. Chua N. H. (2005). MicroRNA directs mRNA cleavage of the transcription factor NAC1 to downregulate auxin signals for arabidopsis lateral root development. Plant Cell 17, 1376–1386. doi: 10.1105/tpc.105.030841 15829603 PMC1091761

[B18] Huang S. Q. Peng J. Qiu C. X. Yang Z. M. (2009). Heavy metal-regulated new microRNAs from rice. J. Inorg. Biochem. 103, 282–287. doi: 10.1016/j.jinorgbio.2008.10.019 19081140

[B19] Juarez M. T. Kui J. S. Thomas J. Heller B. A. Timmermans M. C. P. (2004). microRNA-mediated repression of rolled leaf1 specifies maize leaf polarity. Nature 428, 84–88. doi: 10.1038/nature02363 14999285

[B20] Kozomara A. Birgaoanu M. Griffiths-Jones S. (2019). miRBase: From microRNA sequences to function. Nucleic Acids Res. 47, 155–162. doi: 10.1093/nar/gky1141 PMC6323917 30423142

[B21] Liang G. He H. Yu D. (2012). Identification of nitrogen starvation-responsive microRNAs in arabidopsis thaliana. PloS One 7, e48951. doi: 10.1371/journal.pone.0048951 23155433 PMC3498362

[B22] Liu X. Huang D. Tao J. Miller A. J. Fan X. Xu G. (2014). Identification and functional assay of the interaction motifs in the partner protein OsNAR2.1 of the two-component system for high-affinity nitrate transport. New Phytol. 204, 74–80. doi: 10.1111/nph.12986 25103875 PMC4232926

[B23] Liu Q. Wang F. J. Axtell M. (2014). Analysis of complementarity requirments for plant MicroRNA targeting using a nicotiana benthamiana quantitative transient assay. Plant Cell 26, 741–753. doi: 10.1105/tpc.113.120972 24510721 PMC3967037

[B24] Liu Q. Zhang Y. C. Wang C. Y. Luo Y. C. Huang Q. J. Chen S. Y.. (2009). Expression analysis of phytohormone-regulated microRNAs in rice, implying their regulation roles in plant hormone signaling. FEBS Lett. 583, 723–728. doi: 10.1016/j.febslet.2009.01.020 19167382

[B25] Navarro L. Dunoyer P. Jay F. Arnold B. Dharmasiri N. Estelle M.. (2006). A plant miRNA contributes to antibacterial resistance by repressing auxin signaling. Science 312, 436–439. doi: 10.1126/science.1126088 16627744

[B26] Rogers K. Chen X. (2013). Biogensis, turnover, and mode of action of plant microRNAs. Plant Cell 25, 2383–2399. doi: 10.1105/tpc.113.113159 23881412 PMC3753372

[B27] Sajjad N. Bhat E. A. Shah D. Manzoor I. Noor W. Shah S.. (2021). Nitrogen uptake, assimilation, and mobilization in plants under abiotic stress. Transporters Plant Osmotic Stress 12, 215–233. doi: 10.1016/B978-0-12-817958-1.00015-3

[B28] Srikanth B. Rao I. S. Surekha K. Subrahmanyam D. Voleti S. R. Neeraja C. N. (2016). Enhanced expression of OsSPL14 gene and its association with yield components in rice (*Oryza sativa*) under low nitrogen conditions. Gene 576, 441–450. doi: 10.1016/j.gene.2015.10.062 26519999

[B29] Sullivan C. S. Ganem D. (2005). MicroRNAs and viral infection. Mol. Cell 20, 3–7. doi: 10.1016/j.molcel.2005.09.012 16209940

[B30] Trevisan S. Nonis A. Begheldo M. Manoli A. Palme K. Caporale G.. (2012). Expression and tissue-specific localization of nitrate-responsive miRNAs in roots of maize seedlings. Plant Cell Environ. 35, 1137–1155. doi: 10.1111/j.1365-3040.2011.02478.x 22211437

[B31] Upadhyaya N. M. Surin B. Ramm K. (2000). Agrobacterium-mediated transformation of Australian rice cultivars jarrah and amaroo using modified promoters and selectable markers. J. Plant Physiol. 27, 201–210. doi: 10.1071/PP99078

[B32] Valdés-López O. Yang S. S. Aparicio-Fabre R. Graham P. H. Reyes J. L. Vance C. P.. (2010). MicroRNA expression profile in common bean (Phaseolus vulgaris) under nutrient deficiency stresses and manganese toxicity. New Phytol. 187, 805–818. doi: 10.1111/j.1469-8137.2010.03320.x 20553393

[B33] Vidal E. A. Araus V. Lu C. Parry G. Green P. J. Coruzzi G. M.. (2010). Nitrate-responsive miR393/AFB3 regulatory module controls root system architecture in arabidopsis thaliana. Proc. Natl. Acad. Sci. U.S.A. 107, 4472–4482. doi: 10.1073/pnas.0909571107 PMC2840086 20142497

[B34] Wu K. Wang S. Song W. Zhang J. Wang Y. Liu Q.. (2020). Enhanced sustainable green revolution yield *via* nitrogen-responsive chromatin modulation in rice. Science 367 (6478), eaaz2046. doi: 10.1126/science.aaz2046 32029600

[B35] Xu Z. Zhong S. Li X. Li W. Rothstein S. J. Zhang S.. (2011). Genome-wide identification of MicroRNAs in response to low nitrate availability in maize leaves and roots. PloS One 6, e28009. doi: 10.1371/journal.pone.0028009 22132192 PMC3223196

[B36] Yan M. Fan X. R. Feng H. M. Miller A. J. Shen Q. R. Xu G. H. (2011). Rice OsNAR2.1 interacts with OsNRT2.1, OsNRT2.2 and OsNRT2.3a nitrate transporters to provide uptake over high and low concentration ranges. Plant Cell And Environ. 34, 1360–1372. doi: 10.1111/j.1365-3040.2011.02335.x 21486304

[B37] Yang J. Liu X. Xu B. Zhao N. Yang X. Zhang M. (2013). Identification of miRNAs and their targets using high-throughput sequencing and degradome analysis in cytoplasmic male-sterile and its maintainer fertile lines of brassica juncea. BMC Genomics 14, 9. doi: 10.1186/1471-2164-14-9 23324572 PMC3553062

[B38] Yang Z. Wang Z. Yang C. Yang Z. Li H. Wu Y. (2019). Physiological responses and small RNAs changes in maize under nitrogen deficiency and resupply. Genes Genom. 41, 1183–1194. doi: 10.1007/s13258-019-00848-0 31313105

[B39] Yue E. Li C. Li Y. Liu Z. Xu J. H. (2017). MiR529a modulates panicle architecture through regulating SQUAMOSA PROMOTER BINDING-LIKE genes in rice (*Oryza sativa*). Plant Mol. Biol. 94, 469–480. doi: 10.1007/s11103-017-0618-4 28551765

[B40] Zhang Y. Zhao L. M. Xiao H. Chew J. Xiang J. X. Qian K. Y.. (2020). Knockdown of a novel gene OsTBP2.2 increases sensitivity to drought stress in rice. Genes (Basel) 11, 629. doi: 10.3390/genes11060629 32521717 PMC7349065

[B41] Zhao M. Ding H. Zhu J. K. Zhang F. Li W. X. (2011). Involvement of miR169 in the nitrogen-starvation responses in arabidopsis. New Phytol. 190, 906–915. doi: 10.1111/j.1469-8137.2011.03647.x 21348874 PMC3586203

[B42] Zhao B. Ge L. Liang R. Li W. Ruan K. Lin H.. (2009). Members of miR-169 family are induced by high salinity and transiently inhibit the NF-YA transcription factor. BMC Mol. Biol. 10, 29. doi: 10.1186/1471-2199-10-29 19351418 PMC2670843

[B43] Zhao B. Liang R. Ge L. Li W. Xiao H. Lin H.. (2007). Identification of drought-induced microRNAs in rice. Biochem. Biophys. Res. Commun. 354, 585–590. doi: 10.1016/j.bbrc.2007.01.022 17254555

[B44] Zhou X. F. Wang G. D. Sutoh K. Zhu J. K. Zhang W. X. (2008). Identification of cold-inducible microRNAs in plants by transcriptome analysis. Biochim. Biophys. Acta Gene Regul. Mech. 1779, 780–788. doi: 10.1016/j.bbagrm.2008.04.005 18471443

